# Mesenchymal stem cell-derived exosomes as a new therapeutic strategy in the brain tumors

**DOI:** 10.1186/s13287-022-03212-4

**Published:** 2022-12-20

**Authors:** Elham Ghasempour, Shilan Hesami, Elaheh Movahed, Saeed Heidari keshel, Mohammad Doroudian

**Affiliations:** 1 Department of Tissue Engineering and Applied Cell Sciences, School of Advanced Technologies in Medicine, Shahid Beheshti University of Medical Sciences, Tehran, Iran; 2 Wadsworth Center, New York State Department of Health, Albany, NY USA; 3 Department of Cell and Molecular Sciences, Faculty of Biological Sciences, Kharazmi University, Tehran, Iran

**Keywords:** Exosome, Mesenchymal stem cell, Brain tumor, Blood–brain barrier

## Abstract

Brain tumors are one of the most mortal cancers, leading to many deaths among kids and adults. Surgery, chemotherapy, and radiotherapy are available options for brain tumor treatment. However, these methods are not able to eradicate cancer cells. The blood–brain barrier (BBB) is one of the most important barriers to treat brain tumors that prevents adequate drug delivery to brain tissue. The connection between different brain parts is heterogeneous and causes many challenges in treatment. Mesenchymal stem cells (MSCs) migrate to brain tumor cells and have anti-tumor effects by delivering cytotoxic compounds. They contain very high regenerative properties, as well as support the immune system. MSCs-based therapy involves cell replacement and releases various vesicles, including exosomes. Exosomes receive more attention due to their excellent stability, less immunogenicity and toxicity compare to cells. Exosomes derived from MSCs can develop a powerful therapeutic strategy for different diseases and be a hopeful candidate for cell-based and cell-free regenerative medicine. These nanoparticles contain nucleic acid, proteins, lipids, microRNAs, and other biologically active substances. Many studies show that each microRNA can prevent angiogenesis, migration, and metastasis in glioblastoma. These exosomes can—act as a suitable nanoparticle carrier for therapeutic applications of brain tumors by passing through the BBB. In this review, we discuss potential applications of MSC and their produced exosomes in the treatment of brain tumors.

## Background

Tumor is an uncommon growth of cells and mainly has two types: malignant (cancerous) and benign (noncancerous) [1]. Among the various cancer types, brain tumor caused many deaths in kids and adults [2–5]. In 2016 the brain tumor was the highest tumor-related death in ages 0–14 and the third most common cancer in teenager groups [6]. According to the latest World Health Organization (WHO) report, 700,000 people have been diagnosed with brain tumors. Among these 700,000 cases, 30.1% have diagnosed with malignant tumors. The approximated number of deaths due to brain tumors is 16,830 cases, which is an average survival rate of 35% [4]. Brain tumors can destroy healthy cells and enhance inflammation in the brain. The malignant tumors are divided into two types (a) the first tumor from within the brain itself and (b) the secondary tumor or metastasis that originates from other parts of the body [7–9]. The most common brain tumor is meningioma, gliomas, and glioblastoma multiforme (GBM). Meningiomas are tumors of meninges, managed by surgical and high-risk ones combined with chemotherapy and radiation therapy. Glioblastomas are more prevalent and malignant of the primary tumor that rarely response to treatment [10].

**Fig. 1:**
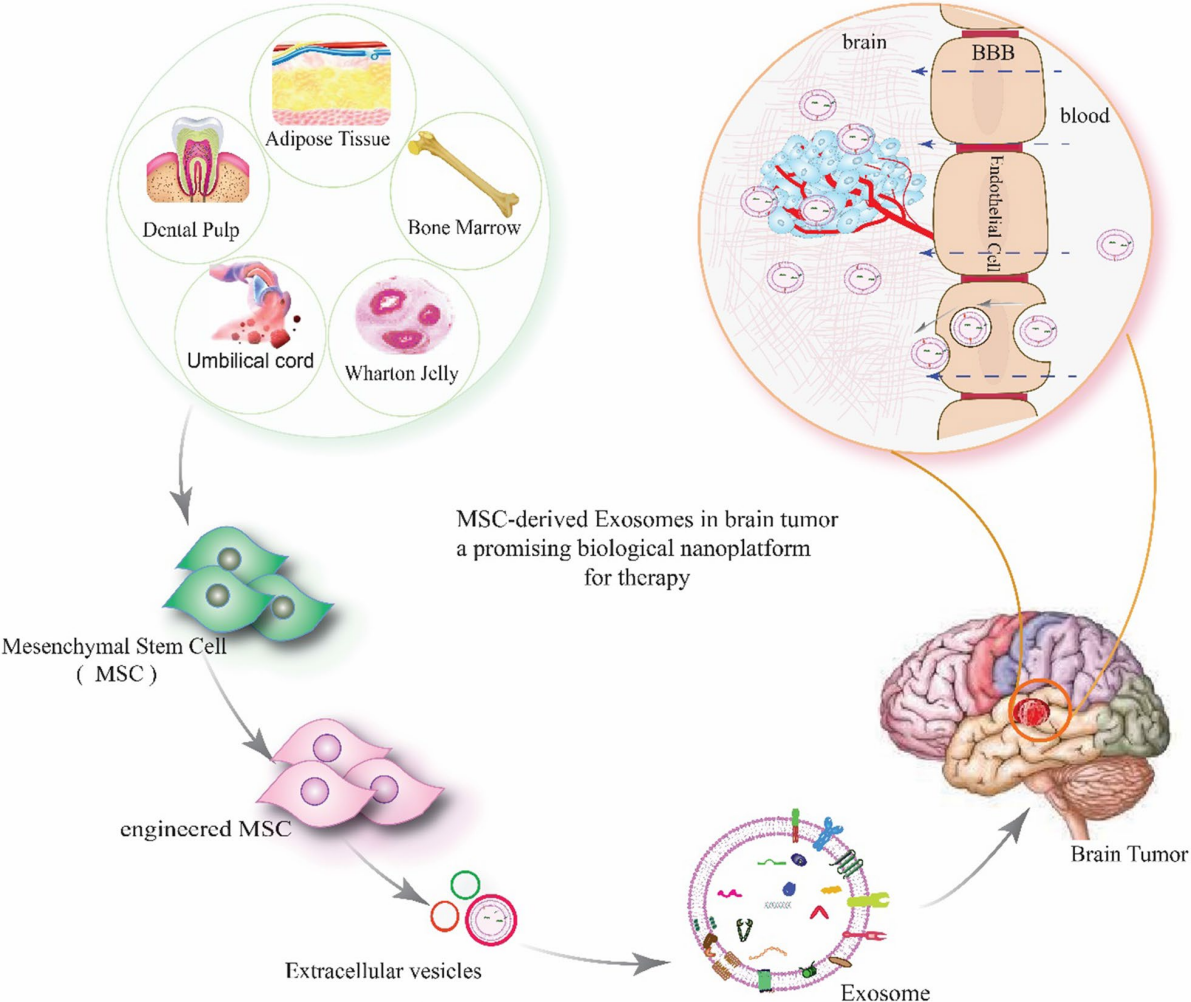
Engineered mesenchymal stem cells (MSCs) derived exosomes inhibit brain tumor progression. The figure was generated by the authors—adobe illustrator version 26.4.1

In spite of the vast progress in cancer treatment, the central nervous system (CNS) tumors have unique features that differentiate them from other neoplasms in the body [11]. Available treatments are not efficient against brain tumors. Because of within brain capillaries, there is a brain–blood barrier (BBB) and endothelial membranes with substantial transendothelial electrical resistance. These barriers strongly control the transcellular and paracellular permeability of molecules in the systemic circulation [11, 12]. Malignant brain tumors such as GBM, the most invasive malignant brain still remain lethal [13, 14]. Thus, there is a necessary need for practical, low-toxicity therapies for brain tumors [14]. Regenerative medicine and cell therapy aim to repair malfunctioning, damaged, and missing tissues, and organs [15]. This review focuses on the characterization of exosomes derived from mesenchymal stem cells (MSCs) and their perspective in cell-free brain tumors therapeutic applications (Fig. 1).

## Regenerative medicine as a new therapeutic strategy in brain tumors

Regenerative medicine is a branch of medicine that in particular has two aims: primary, transferring stem cells into damaged tissues and organs with the maximum safety and efficiency, which may one day replace the transplantation of an entire organ; and secondary, developing strategies to improve the regenerative potential and function of adult stem cells found in various organs [15].

In the last decades, considerable evidence demonstrated that stem cell-based therapies might be hopeful in this field based on the potential therapeutic applications of these cells in damaged organs. Stem cell therapy can be a prospective way for irreversible and incurable diseases [16–18]. Stem cells are undifferentiated cells with self-renewal and multiple differentiation potential that can differentiate into several types of adult cells. Stem cells include induced pluripotent stem cells (iPSCs), embryonic stem cells (ESCs), and adult stem cells. Adult stem cells, also known as resident stem cells or somatic stem cells, are present in small numbers in many adult tissues, such as hematopoietic stem cells (HSC), epithelial stem cells, neural stem cells (NSCs), and mesenchymal stem cells (MSCs) [19].MSCs are one of the most used cell types for regenerative medicine. MSC-based therapies have emerged as a promising strategy in this field [20, 21].

## Mesenchymal stromal/stem cells (MSCs)

MSCs are part of the most crucial pool of adult stem cells that support tissue regeneration under both physiologic and pathologic settings. They contribute to tissue homeostasis, in a dynamic and specialized microenvironment with a distinct design called the "stem cells niche" [22, 23]. Multipotent MSCs present in multiple tissues, including the umbilical cord (UC) [24], bone marrow (BM) [25], adipose tissue (AT) [26], dental pulp [27], placenta [28], skin, heart, lungs, brain, kidneys, thymus, liver, and pancreas. These cells can self-recover and differentiate into multiple tissues, including bone, muscle, cartilage, fat cells, and connective tissue [20].

These cells are immuno-advantaged because of their low expression of CD40, CD80, CD86, major histocompatibility complex I (MHC I), and the lack of MHC II expression, which can be used to treat other organs [29, 30]They reduce encephalitis and restore the blood–brain barrier in the brain (BBB) [31].

MSCs has attracted attention as promising drug carrier for the treatment of brain diseases. For instance, the use of MSCs as a vehicle for targeted gene therapy to the tumor is a novel therapeutic technique [32]. Studies have demonstrated the ability and efficiency of these cells in transferring genes to some tumors such as GBM, breast cancer, and small cell non-cell lung cancer. Due to MSCs strong regenerative activity, immunosuppressive, and immunomodulatory [33, 34]. It seems that applied bone marrow MSC transplantation containing herpes simplex virus thymidine kinase (HSV-TK) gene in combination with prodrug ganciclovir (GCV) would be safe and feasible in the treatment of patients with GBM [32].

One study has indicated that MSC reduced the growth of patient-derived glioma cells and glioma cell lines. Human bone-marrow-derived MSCs impaired endothelial progenitor cell (EPC) to angiogenesis. Phosphorylated Akt, IL-1b, and cathepsin B proteins decreased in co-cultured MSC/glioma through PDGF/PDGFR axis (which has a critical role in angiogenesis). The antitumor effect is mediated by the secretion of soluble factors [35].

MSCs modulate immune function not only via interacting with immune cells such as dendritic cells (DCs), T and B cells, neutrophils, natural killer (NK), and macrophages but also by a powerful paracrine influence [36]. The main active components of paracrine secretion are extracellular vesicles (EVs). Exosomes derived from MSC (MSC-exosomes), have been critically studied to diagnose, prevent, and treat many diseases [37, 38].

## Mesenchymal stem cell-derived exosomes (MSC-E)

Extracellular vesicle (EVs) are lipid‐bilayer vesicles in the 20–1000 nm diameter naturally released from most cell types into the extracellular space [39]. They act as signaling organelles, facilitating intercellular communication by transporting biomolecules like as RNA, proteins, and lipids [40]. EVs are classified into three kinds based on their size and biogenesis. (a) Exosomes are small EVs (40–150 nm in diameter) released by the fusion of multivesicular bodies (MVBs) with the plasma membrane; (b) ectosomes or microvesicles are medium EVs (150–1000 nm in diameter) secreted by the direct budding of the plasma membrane; and (c) apoptosis bodies are random EVs (50–2000 nm in diameter) released during programmed cell death–apoptosis [41, 42]. Among all of EVs, exosomes have attracted much interest in the last decades [43].

**Fig. 2:**
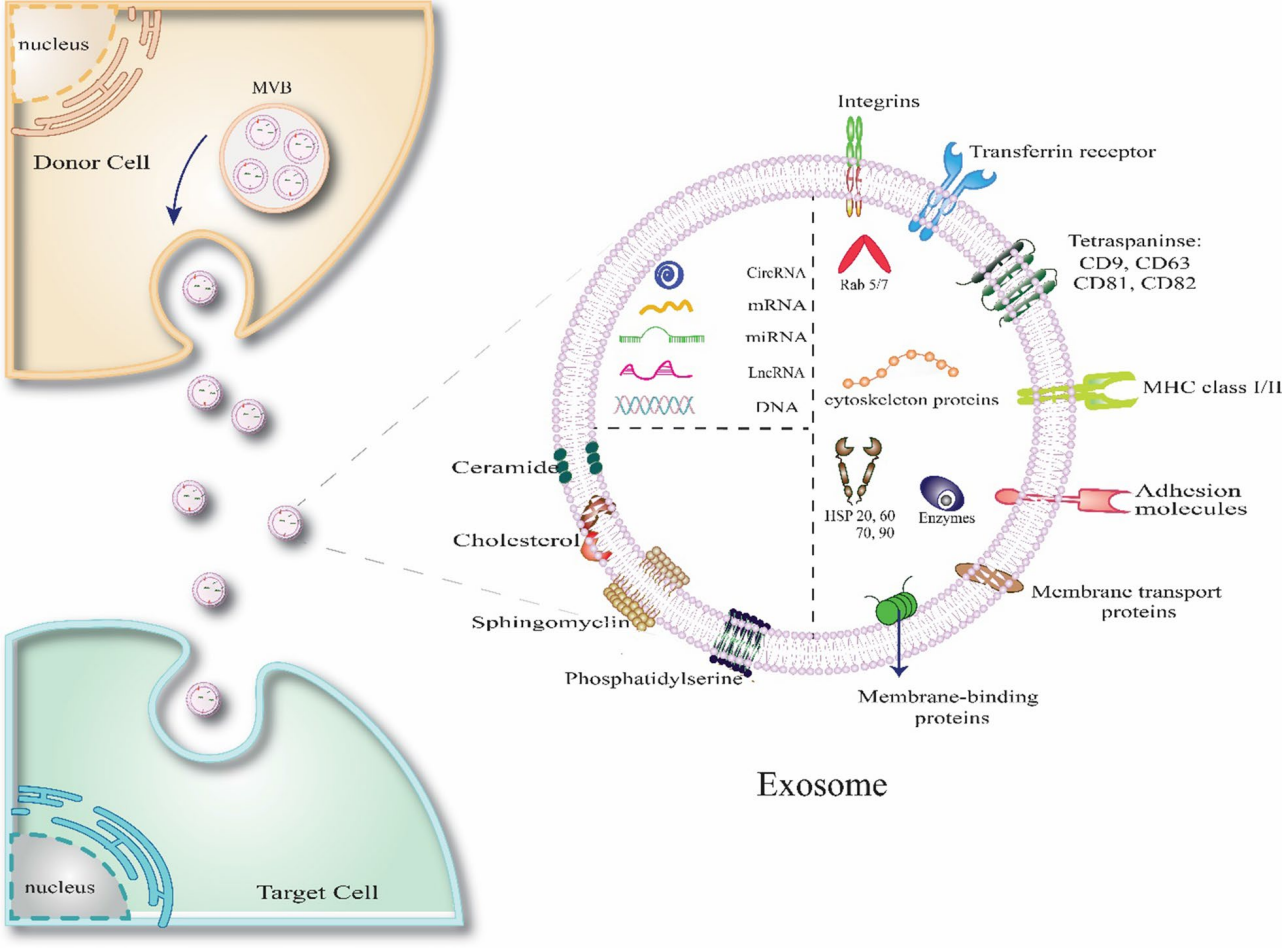
Exosome production and structure. Exosomes are a subtype of the extracellular vesicle with a phospholipid bilayer membrane. These nanoparticles are secreted from cells after the fusion of multivesicular bodies (MVBs or late endosomes) with the plasma membrane. The exosomes contain a wide range of proteins, lipids, mRNAs, RNAs, and DNA molecular cargoes. Tetraspanin family of proteins (CD9, CD63, CD81, and CD82) are common exosome-specific markers. The figure was generated by the authors—adobe illustrator version 26.4.1

Exosomes are a small particle extracellular vesicle that contain protein, nucleic acid, lipid, 5'-nucleotide enzyme etc. [44]. They have a crucial role in biophysical processes in various disease [45, 46]. Environmental factors or pharmacological treatments can modulate the levels of its produced by cultured cells. Due to this reason, its profile alters in different diseases, and the treatment with drugs has alters their content [45]. Exosomes are endosomal origin and contain MHC-I and MHC-II, heat-shock protein, GTPase (EEF1A1, EEF2) membrane-associated protein (CD81, CD82, CD9, and CD63), metabolic enzyme (GADPH, LADH, PKM, aldolase, PGK1), cytoskeletal proteins, and carrier proteins (e.g., albumin) (Fig. 2) [46]. Transportation needs some markers, noticeably CD63 and CD9 [47]. Many studies have demonstrated that people with various cancer have increased exosomes in their blood. The determination cargo of exosomes assists us in diagnostic, pathophysiological, and therapeutic roles; for example, miR-21, miR-219, LRP6, REST1, and cavoline1 increase in their cargo in central nervous system diseases, and in cardiovascular disorder miR-194, miR-133, miR-499, miR-208, miR-34a, miR-192 is up-regulated [44].

Studies have revealed (MVBs) prefer to be in the vicinity of RNA-induced silencing complexes (RISCs). RISC is involved in miRNA biogenesis and plays a key role in miRNA synthesis [48, 49].

MSCs are a rich source of exosomes. MSC-derived exosomes (MSC-E) tend to house damaged tissue, demonstrating that these exosomes like the MSCs from which they are formed, can also home to brain malignancies [50, 51]. According to recent studies MSCs naturally package miRNAs into exosomes. As a result, MSCs could potentially be employed to package exogenous therapeutic miRNAs [52]. Exosomes act as carriers to inhibit tumors by transporting proteins, miRNAs, and chemical drugs. MSC-E contain proteins, mRNAs, miRNAs, and other bioactive molecules. MSC-E proteome contains almost 2000 types of proteins that divide into two groups; First, membrane proteins such as tetraspanins and GPI‐anchored proteins. Second, soluble proteins including signaling proteins, chaperones, heat shock proteins, cytokines, and interleukins [53, 54]. The exosome particle is important for stem cell function because it allows genetic information to be transmitted horizontally between stem cells and tissue-damaged cells. Stem cell-derived Exosomes have features that are similar to those of parent stem cells. It has speculated that stem cell-derived exosomes are a promising treatment approach in regenerative medicine [55]. MSC-E increasingly play an important role in intracellular communication mechanisms, tissue regeneration, and clinical application. With the potential to reduce undesirable side effects and infusional toxicities, uncontrolled cell growth and possible tumor formation may supply significant advantages than live cells [56]. Exosomes are incapable of mutating, duplicating, or causing metastasis. These making exosomes transplantation seem less risky and providing the opportunity for cell uses. These have been tested in different animal models for human diseases. Their functions have been discovered to be quite similar to MSCs. Exosomes are extra-vesical secreted by these cells and create tissue regeneration and cell-free development treatment [43, 57, 58].

## microRNA

MicroRNAs (miRNAs) are non-coding RNAs that play an important role in gene regulation [59]. In human cells, almost 2588 mature miRNAs have been discovered. Some miRNAs are strong tumor suppressors and can target several mRNAs. Deregulation of numerous miRNAs has been linked to a variety of clinical disorders, including benign and malignant tumors, for example GBM [14, 60]. MiRNAs play various roles in disease pathology and physiology. Many cancers have been found to have miRNA dysfunction [61]. Evidence suggests that miRNAs have a role in glioma formation, and that genetically modified MSC can slow the growth of glioma tumors [62]. The regulatory functions of miRNAs have a significant role in GBM by affecting cell proliferation [63], progression [64–66], apoptosis [67], drug resistance [68], and metastasis [69]. It can limit tumor progression by restoring the expression of these miRNAs, indicating a new strategy to tumor therapy [70, 71]. Some microRNAs have been acted as cancer-suppressor [14]. Extracellular exosomes contain many miRNAs, which can be transported from cell to cell by releasing and absorbing exosomes which leads to cross-cellular gene regulation [69]. The effect of MSCs related to secreted considerable amounts of exosomes containing functional miRNAs [72]. It has been also shown that the expression of miR-4731-5p is decreased in glioblastoma. MiR-4731-5p shows an antitumor feature and has been suggested that adipose tissue-derived MSCs (Ad-MSCs) express this miRNA that inhibits GBM cancer cell lines (U87 and U-251) and increases apoptosis in these cells by arresting G1/S phases. miR‐4731 regulates the peripheral myelin protein 22 (PMP22) and CCNA2 (Cyclin A2) genes [67].

## Exosomes are able to cross the blood–brain barrier (BBB)

There are two mechanisms for exosomes to enter the CNS. First, exosomes can absorbed by endothelial cells, then transcytosed into the cell, and transported to the target cell [73]. Secondly, exosomes can pass via endothelial cells' intercellular connections and enter the CNS [74].

Exosome-related miR-105 has been shown to suppress the expression of ZO-1, a crucial molecular component of tight junctions, and thereby remove endothelial cells' barrier function [74]. Exosomal miR-181c has been demonstrated to inhibit 3-phosphoinositide-dependent protein kinase-1 (PDPK1) expression, resulting in lower levels of phosphorylated cofillin and abnormal actin polymerization in brain endothelial cells [75]. In addition, exosomes can cause vascular leakiness, which allows them to access the target tissue [76].

Exosomes released from pathologic cells can change vascular permeability and sometimes BBB integration in degenerative neurological diseases. However, it should be noted that increased BBB permeability is associated with several neurological disorders. More study is still needed to uncover the possible advantages and disadvantages of exosomes' therapeutic potentials with regard to BBB penetrance modulation [76]. Penetration of the BBB and delivery of anti-cancer drugs to the tumor at therapeutic levels is an important challenge in treating brain cancers. Finding a precise delivery approach that is minimally invasive allows maximum efficiency to improve drug delivery across the BBB and the successful treatment of brain cancers. Numerous nanoparticles have been employed for drug delivery, including liposomes, hydrogel, and micelles. However, these particles show critical challenges like high toxicity and low bioavailability. In contrast, exosomes are organotropism, bioavailab, with low toxicity, and low immune responses [77–81].

According to the evidence, Exosomes deliver messages to target cells via at least three mechanisms; first, exosomes can bind to adhesion molecules and receptors on the recipient cell surface with high specificity (without membrane fusion), resulting in receptor activation and a downstream signaling cascade in the target cell [82]. Heparan sulfate (HS) proteoglycans (HSPGs) are surface receptors for exosome adhesion and internalization [83]. Second, exosome content is incorporated into recipient cells following endocytosis. It is the fusion of the exosome membrane with the endosomal membrane. Third, the direct fusion of the exosome membrane with the plasma membrane of the target cells causes their contents to be transferred into these cells’ cytoplasm [82].

## Mesenchymal stem cell-derived exosomes (MSC-E) for brain diseases therapy

Because of MSCs potential to differentiate in neural cells and potent angiomodulatory and immunosuppressive capabilities, they have been described as a new therapeutic agent for treating neurocognitive disorders [84]. A large number of pre-clinical and clinical research have shown that mesenchymal stem cell-derived exosomes (MSC-E) can improve the treatment of neurological diseases relied upon the activity of MSC-sourced bioactive substances (enzymes, lipids, chemokines, cytokines, immunoregulatory proteins, trophic, and growth factors, and miRNAs) which effectively induced neo-angiogenesis, modulated immune response, and increased repair and regeneration of damaged neurons [85].

These neuroprotective and immunosuppressive agents are present within MSC-E, which due to their lipid envelope and nano-sized dimension, easily enter neural tissue and reach the target cells [82]. MSCs function not through a direct cell but via paracrine activity. It has been shown that exosomes are a vital component in regulating the paracrine action of MSCs. Through direct cell membrane fusion, MSC-E delivers its content to recipient cells' cytoplasm and modulates their phenotypic and function [83, 86]. Interaction between MSCs and brain cells induces the production of neurotrophins such as brain-derived neurotrophic factor, nerve growth factor, vascular endothelial growth factor and the anti-inflammatory cytokines that can potentially regenerate nerve growth. This can also stimulate neurologic recovery. Various research studies have demonstrated that the administration of MSCs-E can induce neurogenesis in the hippocampus's subventricular zone (SVZ) and dentate gyrus (DG) and decrease the cognitive impairments associated with traumatic brain injury, stroke, and Parkinson’s disease [76, 87]. MSCs-E are also a part of a therapeutic strategy to treat Alzheimer’s disease by reducing inflammatory activity, promoting neurogenesis, and improving disease-related cognitive deficits [88–90]. MSC-E contain lipids, nucleic acids (4150 miRNAs and 4850 gene products were discovered using microarray analysis and mass spectrometry), proteins (cytokines, chemokines). MiRNAs, notably miR-21 and miR-146, have the potential to modify the phenotypic, function, and viability of neural and immune cells. This is thought to be critical for the beneficial effects of MSC-E in treating neuro-inflammatory disorders [83, 91]. Because there is a minimum side effects associated with the experimental and clinical application of MSCs-E in animals and patients treated with MSC-E. These nano-particles are considered a viable replacement for MSCs in the treatment of degenerative and inflammatory neurological disorders [82].

## Mesenchymal stem cell-derived exosomes (MSC-E) for brain tumors therapy

**Fig. 3:**
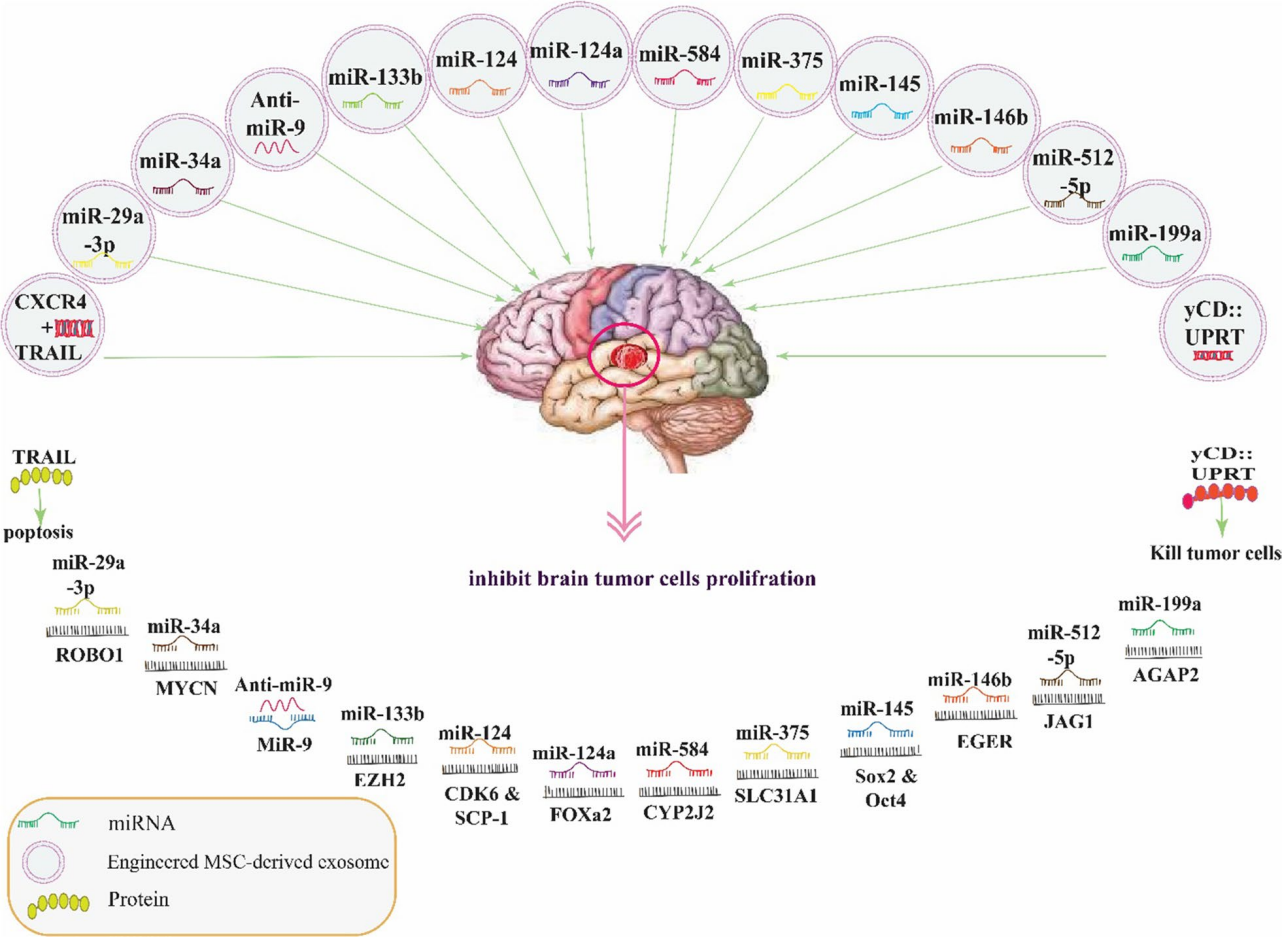
Engineering exosome as a novel Strategy for Treatment. Regulating network of exosomal proteins and miRNAs in brain tumors. The figure was generated by the authors—adobe illustrator version 26.4.1

Engineered MSC-derived exosomes decrease resistance to radiotherapy, chemotherapy, and anti-angiogenesis therapy (Fig. 3). The neurotropic properties of dental pulp-derived MSC (DP-MSCs) are considered in treating a large number of neurological diseases in regenerative medicine. DP-MSCs are known for their high proliferative potential for self-renewability, elasticity, and multi-potential capabilities. Genetically modified human DP-MSCs with yeast uracil phosphoribosyltransferase (UPRT) secrete exosomes can invade and kill glioblastoma cells in the presence of prodrug 5-fluorocytosine (5-FC). The yeast cytosinedeaminase::uracilphosphoribosyltransferase (yCD::UPRT) mRNA was detected in the DP-MSCs exosome’s cargo. Thus, the exosomes internalized in tumor cells acted by translating yCD::UPRT mRNA to enzyme changing 5-FC to 5-FU. The potential of yCD::UPRT-expressing DP-MSCs to convert the relatively non-toxic (5-FC) into the highly toxic antitumor drug 5-fluorouracil (5-FU) combined with their potential to accumulate into tumor sites and micrometastases, making these cells designated therapeutic stem cells (ThSCs) a unique tool for converting prodrugs to cytotoxic drugs directly inside the tumor mass, thus preventing systemic toxicity. Intranasal stem cell delivery is a promising noninvasive method of delivering neural stem/progenitor cells to the brain to treat ischemic brain damage or target intracerebral glioma [92]. The synergistic antitumor study demonstrated that exosomes isolated from MSC^CXCR4+TRAIL^ (exosome^CXCR4+TRAIL^) were essential as a cooperative agent with carboplatin (an anticancer drug) in an MDA-MB-231Br SCID mouse model, potentially creates a new strategy to advance the treatment of breast cancer brain metastases. In human cancer cells, CXCR4 is the most common chemokine receptor. The SDF-1/CXCR4 is important in MSC homing for tumor cell diffusion and metastasis. (TRAIL, also known as Apo2L) can selectively cause apoptosis in tumor cells while causing minimal toxicity in normal cells. This novel use of CXCR4/TRAIL-enriched exosomes to improve chemotherapy efficacy opens up a new avenue for developing a synergistic protocol with anticancer agents to treat brain disease [93].

Glioblastoma stays a fetal tumor with an insignificant outcome (14.5–16.6 month survival rate) an unusual expression of some microRNAs (miR-Let-7f and miR-584-3P) related to this disorder [94, 95] Several studies suggest that MSC-E act as carriers to treat brain tumors by delivering synthetic miRNAs due to MSCs naturally package miRNAs into exosomes. MSCs-E that have been investigated to migrate glioma cells and exert antitumor properties as possible tumor therapy.

MiR-199a is less expressed in glioma tissues than in normal brain tissues, while the GTPase domain, ankyrin repeat and PH domain 2 (AGAP2) are highly expressed. The AGAP2 is a target gene for miR-199a. hMSCs delivered miR-199a to the glioma cells via the exosomes, which suppressed glioma cell proliferation, migration, and invasion. They also increased temozolomide chemosensitivity and reduced tumor development in vivo. It has been shown that miR-199a, when transported by hMSCs derived exosomes, can negatively influence AGAP2 expression, thus preventing proliferation and increasing glioma cell apoptosis. There is still insufficient evidence to support the efficient delivery of miR-199a from MSCs to glioma cells via exosomes. In conclusion, additional research is needed to validate and substantiate our findings from this study [62].

MiR-512-5p and Jagged1 (JAG1) expression patterns, as well as their interactions in glioblastoma, were studied. In 2021 a study showed that bone marrow stem cell (BMSC)-derived exosomes via transporting miR-512-5p alter tumor phenotypes. In glioblastoma tissue and cells, miR-512-5p was downregulated, and its target gene is JAG1. JAG1 expression is increased by silencing miR-512-5p. JAG1 expression in GBM is inhibited by BMSC-exosomal miR-512-5p. JAG1 silencing has been shown to reduce cyclin D1 expression. These findings shed light on the molecular therapy mechanism for GBM treatment and highlight the potential for BMSC-Exo to transport miR-512-5p into GBM [70]. Moreover, cyclin D1 regulates the cell cycle by binding to cyclin-dependent kinase (CDK4, CDK6). miR-512-5p by targeting JAG1 reduces G1-arrest-relevant cell cycle regulators to prevent GBM development. Exosome therapy in the long term will include merging targeted exosomes with anticancer drugs [70, 96].

One of the miRs that express very high in common brain tissue constraint the glioma is miR-29a-3p. One in-vitro study demonstrated miR-29a-3p inhibits migration of glioma cells and their angiogenesis. Roundabout homolog 1 protein, encoded by Roundabout Guidance Receptor 1(ROBO1) is the target of this microRNA. To take advantage of miR-29a-3p's anti-tumor effects, they modified MSCs to act as a "bio-factory" for exosomes expressing miR-29a-3p [97].

One study to show MSCs can release synthetic miRs uses MSCs that have been investigated to migrate glioma cells and exert antitumor properties as possible tumor therapy. The- tested MSCs derived from placenta, umbilical cord, adipose tissue, and bone marrow which can carry synthetic miRNAs to the tumor environment. Using flow cytometry and in situ hybridization showed MSCs delivered miR-145 and miR-124 to co-culture medium via releasing exosomes. This delivered miR-145 and miR-124 notably decreased the migration of glioma cells by targeting Synaptonemal Complex Protein 1 (SCP-1) and SRY-Box Transcription Factor 2 (Sox2) genes. Antisense, siRNA, and miRNA small RNAs are emerging as promising therapeutic agents for various diseases. The efficient delivery of these molecules is critical to their clinical success [98]. The other study demonstrated that miR-146b expressed lower in GBM and transfected MSCs with exosome of miR-146b then injected intra-tumor. The study showed miR146b decreased motility and invasion of glioma cells, and its target being Epidermal Growth Factor Receptor (EGFR) mRNA. EGFR gene was duplicated in 40% of glioblastoma multiforme and increased its related glioma invasiveness and malignancy. MiR-146b decreased expression of EGFR, glioblastoma cell migration and viability. These findings imply that exporting specific therapeutic mRNA into MSC exosomes can be a new treatment strategy for malignant glioma [52]. The roles of human marrow stromal cells (hMSCs) in glioblastoma growth are still argumentative. In 2018, Sheng-Ze Deng in 2018 analyzed the effect of these cells and their regulatory hMSC exosomal miR-375 in glioblastoma. They demonstrated that exosomal miR-375 decreased the progression of glioma cells through suppress Solute Carrier Family 31 Member 1 (SLC31A1) and is suitable for the treatment of glioma. Thus, serve as a promising novel drug delivery method and target for developing therapeutic modalities against gliomas [99].

MSC can release exosomes, and these cells have a critical role in the promotion of glioblastoma. In vivo studies used mice injected with U87 cells and then exposed MSC-derived exosomes. MiR-584 suppresses various cancer include glioblastoma, by attaching to 3-UTR on Cytochrome P450 Family 2 Subfamily J Member 2 (CYP2J2) reduced proliferation and invasion of glioblastoma cells. They show that miRNA merge into MSC-derived exosomes with glioblastoma cells. These exosomes contain miR-584 that could modulate tumor development. These discoveries provide a new approach to therapy in glioblastoma cancer. Malignant gliomas were reduced after exposure to exosomes derived from miRNA-584 transfected MSCs; the treatment did not affect the animals' body weight [100].

Frederick M. Lang et al. demonstrated that ex-vivo bone-marrow-derived MSCs could pack miRs in the engineered exosome and deliver to glioma tumors. Western blotting, electron microscopy, and Nanosight techniques showed that the isolated vesicles were exosomes. These particles containing miR-124a showed significantly decreased viability and clonogenicity of tumor cells. In-vitro and in-vivo studies showed that miR-124a by silencing FOXa2 malapropos the lipid accumulation and have anti-glioma properties. As a result, it supports the idea that miR-124a downregulation of FOXA2 reduces GSC viability due to an induced inability to utilize lipids [101].

Sharif et al. [102] demonstrated that Wharton's jelly-MSCs (WJ-MSCs) have the potency to transfer microRNAs to glioblastoma cells. They showed that through a dependent or exosome-independent process, miR-124 was delivered with WJ-MSCs to U87. Delivered miR-124 reduced the luciferase activity of the CDK6 gene. Additionally, increased the chemosensitivity to temozolomide and reduced the migration of glioblastoma cells. As an outcome, combining WJ-MSCs with delivered miR-124 and TMZ can be a new and effective treatment for GBM cancer. The other study received findings that showed exosomes from MSC carrying miR-133b decreased glioma size through the Wnt/*β*-catenin pathway by silencing Enhancer of Zeste 2 Polycomb Repressive Complex 2 Subunit (EZH2). In glioma tissues, MiR-133b was downregulated, but EZH2 was increased. Therefore, silencing EZH2 caused decreased proliferation, migration, and invasion of glioma cell. U87 were co-cultured with MSCs to investigate the pattern of miR-133b and EZH2 using RT-qPCR. These findings demonstrated that MSC-derived exosomes transferring miR-133b into glioma cells could potentially inhibit EZH2 expression by blocking the Wnt/*β*-catenin signaling pathway, thereby suppressing glioma cell proliferation, migration, and invasion [103]. Munoz et al. [104] publications reported that miR-9 in tumor with TMZ-resistant increase. MiR-9 was rolled in the expression of p-glycoprotein (drug transporter), and Anti-miR-9 reversed the expression of drug transporter. It also sensitized the glioma cell to temozolomide, as demonstrated increased caspase activity and cell death. They investigated the influential role of MSCs in the delivery of synthetic anti-miR-9 to increased chemo resistance of glioma cells by extra vesicle including exosomes. They discovered that secreted exosomes play a role in MSC-GBM cell communication.

One study demonstrates the effect of hBMSC released exosomes containing miR-34a. The efficacy of exosomal miR-34a in nude mice with GBM cells was detected. The result showed that miR-34a in exosome from hBMSC negatively regulate the N-myc proto-oncogen or basic helix-loop-helix protein 37 (MYCN) in GBMs cell and reduce their proliferation, migration, invasion, and, finally, tumorigenesis in-vitro and in-vivo experimentation [105]. Glioma stem cells (GSCs) have been linked to resistance to radiotherapy and chemotherapy [106, 107].The main reasons for the poor treatment outcome are issues accurately delivering therapeutic agents to the target GBM brain tumors. Liposomes and viral vectors currently used for miR delivery are unsuitable due to low efficiency and safety [108]. MSCs-E can deliver peptides [109], prodrugs [109, 110], oncolytic viruses, and miRNA mimics to glioma cells and GSCs in culture, as well as glioma xenografts in vivo. These synthetic miRNA mimics can function as physiologically functional molecules, silencing genes in ways similar to cellular miRNAs. As a result, based on these findings, MSCs-E provide a novel approach for the targeted delivery of anti-cancer cargoes [99]. Despite the importance of this issue, clinical trials on MSC-E in the field of brain disease are limited. An Open-Label Clinical Trial titled exosome derived from allogeneic mesenchymal stem cells in patients with acute ischemic stroke in Phase I/II (ClinicalTrials.gov identifier: NCT03384433) purpose to explore improving these patients who received MSC-E enriched by miR-124 through Stereotaxis/Intraparanchymal one month after the attack. Another single-center, open-label Clinical Trial titled the Safety and the Efficacy Evaluation of Allogenic Adipose MSC-E in Patients with Alzheimer's Disease in Phase I/II (ClinicalTrials.gov identifier: NCT04388982) aims to investigate the safety and efficacy of the allogenic adipose MSCs-E in the treatment of mild to moderate dementia caused by Alzheimer's Disease. The results of these studies are not posted on ClinicalTrials.gov.

## Conclusion

Brain cancer treatment is still one of the biggest challenges in oncology [111]. Among different methods for diagnoses, MRI has a common noninvasive technique because it has high resolution and high helpful contrast for soft tissue [106, 107]. The prospect and therapy approach for primary brain tumors remained uncleared, despite drug findings and anticancer therapy improvement [108]. The process of drug delivery and efficient treatments are the main-goals [109]. EVs regulate cell-to-cell communication. EVs encompass mRNA, proteins, and miRNA. They transfer functional molecules to the side of cells [110, 112]. Exosomes are nanoparticles with the therapeutic feature for increase antitumor and drug delivery [113]. Currently, there is evidence demonstrating that exosomes produced by various stem cell sources, particularly MSCs, have neurotherapeutic future and can be successfully used to treat several brain tumors [114]. These particles impact progress tumor development and modulate the main properties of the tumor by delivery of spatial microRNAs [115]. MSCs are immense in regeneration, but these cells have a contradictory effect on tumor development [116, 117].

Despite significant efforts to use exosomes as targeted therapeutic carriers in clinical applications, there are substantial challenges that future research should address. A significant limiting factor is the lack of a standardized method for isolating and purifying an enriched population of exosomes. Conventional isolation techniques necessitate multiple steps of ultracentrifugation. However, these methods are time-consuming, and obtained exosomes are frequently contaminated with non-exosomal EVs. As the exosome-based drug/gene delivery systems, the presence of other types of EVs will reduce therapeutic efficiencies.

Another limiting factor is their low production of exosomes which limits their therapeutic potential. Various methods have been developed to increase exosomes’ therapeutic use including engineering, genetic manipulation, and three-dimensional culture (3DDC) of these cells. According to new studies, exosomes produced by MSC in 3DCC spheroids increased significantly compared to Two-dimensional culture (2DCC)-derived exosomes. Third, cell culture-derived exosomes can vary and exhibit inconsistent properties even when the same type of donor cells are used. As a result, precise and efficient characterization studies of exosomes are required before using them as therapeutic carriers [118–120]. It is better to continue future studies with caution and focus on modifying the exosomes to precisely orient them toward the desired target, as it may lead to optimal therapeutic outcomes with minimal side effects.

## Data Availability

Not applicable.

## Abbreviation

BBB: Blood–brain barrier
MSCs: Mesenchymal stem cells
WHO: World Health Organization
GBM: Glioblastoma multiforme
MRI: Magnetic resonance imaging
CNS: Central nervous system
iPSCs: Including induced pluripotent stem cells
ESCs: Embryonic stem cells
HSC: Hematopoietic stem cells
NSCs: Neural stem cells
UC: Umbilical cord
BM: Bone marrow
AT: Adipose tissue
MHC I: Histocompatibility complex I
HSV-TK: Herpes simplex virus thymidine kinase
GCV: Ganciclovir
EPC: Endothelial progenitor cell
DCs: Dendritic cells neutrophils
NK: Natural killer
EVs: Extracellular vesicles
MVBs: Multivesicular bodies
MSC-exosomes: Exosomes derived from MSC
RISCs: RNA-induced silencing complexes
miRNAs: MicroRNAs
Mesenchymal stem cell-derived exosome MSC-E(PDPK1): 3-Phosphoinositide-dependent protein kinase-1
SVZ: Subventricular zone
DG: Dentate gyrus
DP-MSCs: Dental pulp-derived MSC
UPRT: Uracil phosphoribosyltransferase
5-FC: 5-Fluorocytosine
5-FU: 5-Fluorouracil
yCD::UPRT: Yeast cytosinedeaminase::uracilphosphoribosyltransferase
ThSCs: Therapeutic stem cells
AGAP2: GTPase domain, ankyrin repeat and PH domain 2
JAG1: Jagged1
BMSC: Bone marrow stem cell
ROBO1: Roundabout Guidance Receptor 1
SCP-1: Synaptonemal Complex Protein 1
Sox2: SRY-Box Transcription Factor 2
EGFR: Epidermal Growth Factor Receptor
hMSCs: Human marrow stromal cells
SLC31A1: Solute Carrier Family 31 Member 1
CYP2J2: Cytochrome P450 Family 2 Subfamily J Member 2
WJ-MSCs: Wharton's jelly-MSCs
EZH2: Enhancer Of Zeste 2 Polycomb Repressive Complex 2 Subunit
MYCN: *N*-Myc proto-oncogen or basic helix-loop-helix protein
